# Relationship between changes in BNP, PCT, and SIGIRR levels in peripheral blood and prognosis of sepsis patients caused by abdominal infection and construction of a nomogram

**DOI:** 10.1097/MD.0000000000048227

**Published:** 2026-04-17

**Authors:** Shangzhen Li, Yunyun Cui, Jiaqi Yuan, Xiankui Wang, Naixi Ji, Jian Zhang, Changqing Yao, Haiyun Zhang

**Affiliations:** a Department of Critical Care Medicine, Xining Second People’s Hospital, Xining, Qinghai Province, China; b Department of Gastroenterology, Xining Second People’s Hospital, Xining, Qinghai Province, China.

**Keywords:** abdominal infection, brain natriuretic peptide, myocardial injury, procalcitonin, sepsis

## Abstract

This study aimed to explore the changes of serum brain natriuretic peptide (BNP), procalcitonin (PCT), and recombinant single Ig IL-1-related receptor (SIGIRR) in patients with sepsis associated with abdominal infection in emergency and their relationship with prognosis and construct a nomogram prediction model. A total of 160 emergency patients with abdominal infection were divided into simple infection group (n = 80) and sepsis group (n = 80) according to whether sepsis occurred or not, and the baseline data, serum BNP, PCT, and SIGIRR levels of the 2 groups were compared. Patients in the sepsis group were divided into survival group (n = 52) and death group (n = 28) according to their survival conditions within 28 days. The baseline data, serum BNP, PCT, and SIGIRR levels of the 2 groups were compared, and the diagnostic value of serum BNP, PCT, and SIGIRR levels in the sepsis group was analyzed by receiver operating characteristic (ROC) curve. The related factors affecting the prognosis of sepsis patients were analyzed using a binary logistic equation. The nomogram risk prediction model of poor prognosis of sepsis patients is drawn and verified by calibration curve and ROC curve. The serum levels of BNP, PCT, and SIGIRR in the simple infection group were significantly lower than those in the sepsis group (*P* < .05). In patients with sepsis group, the serum levels of BNP, PCT, and SIGIRR in the death group were significantly higher than those in the survival group (*P* < .05). ROC curve analysis showed that the areas under the curve of serum BNP, PCT, and SIGIRR in predicting the poor prognosis of sepsis patients were 0.874, 0.824, and 0.784, respectively. Univariate and multivariate logistic regression analyses showed that serum BNP, PCT, and SIGIRR levels were independent risk factors for poor prognosis of sepsis patients (*P* < .05). Based on the independent risk factors (BNP, PCT, and SIGIRR) screened by logistic regression model, the nomogram was constructed, with Concordance Index of 0.947, which indicated that the nomogram had good discrimination and consistency. The levels of BNP, PCT, and SIGIRR of patients with sepsis were significantly increased, which was significantly related to the poor prognosis of patients. The nomogram prediction model constructed on this basis is helpful for clinicians to provide better individualized treatment for the poor prognosis of patients with sepsis associated with abdominal infection.

## 1. Introduction

Sepsis refers to a clinical syndrome in which the host response induced by pathogen infection is out of balance, which leads to serious organ dysfunction. The mortality rate of this syndrome is high. According to a report on the intensive care units (ICU) of 44 hospitals in China, the incidence of sepsis in ICU is 20.6%, which greatly affects the health of people.^[1]^ Abdominal infection is a common factor of sepsis. Abdominal infection-related sepsis accounts for 26.57% of the total cases of sepsis, second only to pulmonary infection. It is usually due to local or diffuse abdominal infection caused by the rupture of the gastrointestinal tract or abdominal organs. Most patients show symptoms such as abdominal pain, abdominal distension, and vomiting. Early clinical symptoms are very similar to those of an ordinary abdominal infection, and there is a certain risk of missed diagnosis. If patients are not diagnosed and treated in time, there may be a risk of septic shock or death. Therefore, early diagnosis and prevention are positive for improving the prognosis of patients.^[2]^

Acute infection is the most common signal of sepsis, and procalcitonin (PCT) is a pro-peptide, which is the most common indicator of bacterial infection. When it is >0.5 ng/mL, it indicates that patients need anti-infection treatment, which is a common marker of sepsis, and many experts at home and abroad recommend it as a landmark indicator of sepsis infection.^[3]^ Brain natriuretic peptide (BNP) is a natural hormone with diuretic and vasodilating effects, and it is a classic index for evaluating myocardial injury. Recent studies have found that BNP has certain evaluation value for the prognosis of sepsis, which may be related to acute myocardial dilatation in septic patients, but it needs further clinical confirmation.^[4]^ Recombinant single Ig IL-1-related receptor (SIGIRR) is a newly discovered infection marker, which is widely expressed in major human tissues and organs. Studies have shown that it can regulate the outcome and prognosis of infectious diseases and negatively regulate the immune response.^[5]^ In this study, the correlation and predictive value of serum BNP, PCT, and SIGIRR levels with sepsis associated with abdominal infection were analyzed; the risk factors of poor prognosis of sepsis associated with abdominal infection were analyzed; and a nomogram risk prediction model was constructed, aiming at effectively guiding clinical diagnosis and treatment.

## 2. Materials and methods

### 2.1. Data collection

A total of 160 cases of emergency abdominal infection admitted to our hospital from January 2020 to October 2023 were included in this retrospective observational study. The inclusion criteria are as follows: meet the diagnostic criteria of *Guidelines for Diagnosis and Treatment of Abdominal Infection in China (2019 Edition*),^[6]^ and be diagnosed as acute abdominal infection by routine blood examination, B-ultrasound, and abdominal computed tomography examination; are between 18 and 80 years old; and have complete clinical data. The following are the exclusion criteria: sepsis associated with other organ infections; combined with malignant tumor; severe failure of liver and kidney function; autoimmunity is defective; mental abnormality or noncooperation with treatment; uncertain prognosis indicators; missing data; incomplete treatment (given up treatment); and unknown infection source. This study was conducted according to the ethical principles of medical research involving human subjects in the Helsinki Declaration. Patients and their families signed informed consent. All the patients were divided into simple infection group (n = 80) and sepsis group (n = 80) according to the diagnostic criteria in *Guidelines for Emergency Treatment of Sepsis/Septic Shock in China (2018*),^[7]^ including 47 males and 33 females in the simple infection group; the average age was 58.84 ± 7.23 years. There were 44 males and 36 females in the sepsis group. The average age was 59.70 ± 5.37 years.

### 2.2. Definitions of key terms

Simple infection group: patients diagnosed with acute abdominal infection who met the diagnostic criteria of *Guidelines for Diagnosis and Treatment of Abdominal Infection in China (2019 Edition*),^[6]^ without meeting the diagnostic criteria of sepsis. The clinical manifestations were limited to local abdominal inflammatory responses (such as abdominal pain, abdominal distension, and local tenderness), without systemic inflammatory response syndrome (SIRS) and organ dysfunction related to infection.

Sepsis group: patients who met the diagnostic criteria of *Guidelines for Emergency Treatment of Sepsis/Septic Shock in China (2018*),^[7]^ specifically, on the basis of confirmed abdominal infection, patients presented with SIRS accompanied by organ dysfunction, tissue hypoperfusion, or hypotension. SIRS was defined as meeting at least 2 of the following criteria: body temperature > 38°C or <36°C; heart rate > 90 beats/min; respiratory rate > 20 breaths/min or arterial partial pressure of carbon dioxide (PaCO_2_) < 32 mm Hg; and white blood cell (WBC) count > 12 × 10^9^/L, <4 × 10^9^/L, or the proportion of immature neutrophils > 10%. Organ dysfunction was evaluated by sequential organ failure assessment (SOFA) score (an increase of ≥2 points from the baseline indicated the presence of organ dysfunction related to sepsis).

### 2.3. Observation index

All patients received routine examination and treatment after admission, and the clinical baseline data of patients were counted, including gender, age, body mass index (BMI), pathogen type, WBC count, C-reactive protein (CRP), oxygenation index, albumin (ALB), mechanical ventilation time, ICU stay, acute physiology and chronic health evaluation II (APACHE II) score,^[8]^ and SOFA score.^[9]^ Among these indicators, WBC and ALB were detected by drawing patients’ venous blood, centrifuging to separate the supernatant, and using the BH-6680 automatic hematology analyzer; CRP was detected by drawing venous blood, centrifuging to separate the supernatant, and using an enzyme-linked immunosorbent assay (ELISA) biochemical detection kit. The APACHE II score ranges from 0 to 71, with higher scores indicating more severe patient conditions; the SOFA score ranges from 0 to 20, with higher scores indicating more severe organ failure in patients.

Detection of serum BNP, PCT, and SIGIRR levels: 5 mL of venous blood was drawn from each patient and placed in an anticoagulant tube. The supernatant was separated by centrifugation at 3000 rpm and stored in a −80°C refrigerator. The levels of BNP, PCT, and SIGIRR in serum were detected using an ELISA biochemical detection kit.

Prognostic evaluation: Patients in the acute abdominal infection combined with sepsis group were divided into the survival group (n = 52) and the death group (n = 28) based on their survival status within 28 days. The 28-day survival time was selected as the prognostic evaluation time frame because it is a widely recognized and standardized time point in clinical sepsis research. As recommended by international guidelines (such as the Surviving Sepsis Campaign guidelines) and most relevant clinical studies, 28 days can fully reflect the short-term prognosis of sepsis patients, covering the critical period of disease progression, treatment response, and organ function recovery. It also facilitates the comparison of research results with other similar studies.

### 2.4. Statistical analysis

SPSS (version 26.0) and R software (version 4.2.1) were used to analyze the data statistically. The categorical data were expressed as n (%), and the comparisons between groups were performed using the chi-square test or Fisher exact test (when n < 5). The continuous data were expressed as mean ± standard deviation (*x̄* ± *s*), and comparisons between groups were conducted using the *t* test. The receiver operating characteristic (ROC) curve was used to evaluate the predictive value of serum BNP, PCT, and SIGIRR levels on the poor prognosis of the sepsis group. Binary logistic regression analysis was used to analyze the influencing factors of poor prognosis of sepsis patients. According to the results of logistic regression multivariate analysis, R software (version 4.2.1) was used to establish a nomogram prediction model, and the prediction accuracy and discrimination ability of the model were determined by calibration curve, ROC curve, and consistency index (C-index), with α = 0.05 as the test level.

## 3. Results

### 3.1. Comparison of baseline data between simple infection group and sepsis group

There was no significant difference in gender, age, BMI, and pathogen type between the simple infection group and the sepsis group (*P* > .05). Specifically, the male ratio was 58.75% (47/80) in the simple infection group and 55.00% (44/80) in the sepsis group; the average age of the simple infection group was 58.84 ± 7.23 years, and that of the sepsis group was 59.70 ± 5.37 years; the mean BMI was 20.47 ± 0.99 kg/m^2^ and 20.60 ± 1.21 kg/m^2^ in the simple infection group and sepsis group, respectively; the main pathogen types in both groups were *Escherichia coli*, *Klebsiella pneumoniae*, and *Enterococcus faecalis*, with no statistical difference in distribution (*P* > .05). However, there were significant differences in WBC, CRP, ALB, APACHE II, and SOFA between the 2 groups (*P* < .05). The simple infection group had a lower WBC count (10.11 ± 2.03 × 10^9^/L vs 14.03 ± 3.15 × 10^9^/L), lower CRP level (68.26 ± 10.28 mg/L vs 94.61 ± 18.96 mg/L), higher ALB level (33.25 ± 4.44 g/L vs 27.53 ± 4.03 g/L), lower APACHE II score (22.15 ± 2.45 points vs 24.35 ± 5.11 points), and lower SOFA score (4.28 ± 0.64 points vs 9.21 ± 1.67 points) compared with the sepsis group. The detailed data are shown in Table 1.

**Table 1:** Comparison of baseline data between simple infection group and sepsis group.

Indicator	Simple infection group (n = 80)	Sepsis group (n = 80)	*t*/χ^2^	*P*
Age (yr)	58.84 ± 7.23	59.70 ± 5.37	−0.856	.393
BMI (kg/m^2^)	20.47 ± 0.99	20.60 ± 1.21	−0.693	.489
Gender			0.229	.632
Male	47 (58.75%)	44 (55.00%)		
Female	33 (41.25%)	36 (45.00%)		
Pathogen type			5.829	.120
G+	18 (22.50%)	9 (11.25%)		
G−	35 (43.75%)	37 (46.25%)		
Fungus	9 (11.25%)	6 (7.50%)		
Mixed infection	18 (22.50%)	28 (35.00%)		
WBC (×10^9^/L)	10.11 ± 2.03	14.03 ± 3.15	−9.328	<.001
CRP (μg/L)	68.26 ± 10.28	94.61 ± 18.96	−6.898	<.001
ALB (g/L)	33.25 ± 4.44	27.53 ± 4.03	8.530	<.001
APACHE II score (points)	22.15 ± 2.45	24.35 ± 5.11	−3.473	<.001
SOFA score (points)	4.28 ± 0.64	9.21 ± 1.67	−24.661	<.001

ALB = albumin, APACHE II = acute physiology and chronic health evaluation II, BMI = body mass index, CRP = C-reactive protein, G+ = gram-positive, G− = gram-negative, ICU = intensive care unit, SOFA = sequential organ failure assessment, WBC = white blood cell.

### 3.2. Comparison of serum BNP, PCT, and SIGIRR levels between simple infection group and sepsis group

The levels of serum BNP, PCT, and SIGIRR in the simple infection group were significantly lower than those in the sepsis group (*P* < .05), as shown in Table 2.

**Table 2:** Comparison of serum indicators between simple infection group and sepsis group.

Groups	BNP (pg/mL)	PCT (ng/mL)	SIGIRR (pg/mL)
Simple infection group	125.31 ± 19.32	2.62 ± 0.38	70.14 ± 16.61
Sepsis group	469.74 ± 90.94	8.90 ± 2.16	81.14 ± 16.15
*t*	−33.137	−25.584	−4.246
*P*	<.001	<.001	<.001

BNP = brain natriuretic peptide, PCT = procalcitonin, SIGIRR = recombinant single Ig IL-1-related receptor.

### 3.3. Comparison of baseline data between survival group and death group in sepsis patients

There was no significant difference in gender, age, BMI, pathogen type, and WBC between the survival group and the death group (*P* > .05), but there were significant differences in CRP, ALB, APACHE II, SOFA, oxygenation index, mechanical ventilation time, and ICU stay between the 2 groups (*P* < .05), as shown in Table 3.

**Table 3:** Comparison of baseline data of patients with acute abdominal infection-related sepsis with different prognosis status.

Indicator	Survival group (n = 52)	Death group (n = 28)	*t*/χ^2^	*P*
Age (yr)	59.33 ± 5.34	60.39 ± 5.47	−0.845	.401
BMI (kg/m^2^)	20.53 ± 1.23	20.72 ± 1.17	−0.671	.504
Gender			0.036	.851
Male	29 (55.77%)	15 (53.57%)		
Female	23 (44.23%)	13 (46.43%)		
Pathogen type			0.352	.950
G+	6 (11.54%)	3 (10.71%)		
G−	25 (48.08%)	12 (42.86%)		
Fungus	4 (7.69%)	2 (7.14%)		
Mixed infection	17 (32.69%)	11 (39.29%)		
WBC (×10^9^/L)	14.25 ± 3.37	13.62 ± 2.72	0.856	.395
CRP (μg/L)	75.48 ± 17.26	130.14 ± 23.01	−11.994	<.001
ALB (g/L)	28.53 ± 3.99	25.67 ± 3.45	3.195	.002
APACHE II score (points)	23.14 ± 4.92	26.57 ± 4.78	−3.001	.004
SOFA score (points)	8.54 ± 0.64	10.46 ± 2.22	−5.849	<.001
Oxygenation index (mm Hg)	285.79 ± 37.89	231.14 ± 34.42	6.348	<.001
Mechanical ventilation time (h)	56.23 ± 7.20	65.36 ± 10.94	−4.487	<.001
ICU stay (d)	7.02 ± 1.29	9.82 ± 1.28	−9.293	<.001

ALB = albumin, APACHE II = acute physiology and chronic health evaluation II, BMI = body mass index, CRP = C-reactive protein, G+ = gram-positive, G− = gram-negative, ICU = intensive care unit, SOFA = sequential organ failure assessment, WBC = white blood cell.

### 3.4. Comparison of serum BNP, PCT, and SIGIRR levels between survival group and death group in sepsis patients

The serum levels of BNP, PCT, and SIGIRR in the death group were significantly higher than those in the survival group (*P* < .05), as shown in Table 4.

**Table 4:** Comparison of serum indicators between simple infection group and sepsis group.

Groups	n	BNP (pg/mL)	PCT (ng/mL)	SIGIRR (pg/mL)
Survival group	52	432.56 ± 46.57	7.96 ± 0.94	75.95 ± 15.50
Death group	28	538.79 ± 111.77	10.64 ± 2.68	90.78 ± 12.71
*t*		−5.980	−6.521	−4.335
*P*		<.001	<.001	<.001

BNP = brain natriuretic peptide, PCT = procalcitonin, SIGIRR = recombinant single Ig IL-1-related receptor.

### 3.5. Univariate and multivariate logistic regression analysis of the relationship between serum BNP, PCT, SIGIRR levels and prognosis of sepsis patients

Univariate and multivariate logistic regression analysis (death group = 1, survival group = 0) showed that serum BNP, PCT, and SIGIRR were independent risk factors for poor prognosis of sepsis patients (*P* < .05), as shown in Table 5.

**Table 5:** Correlation analysis between serum BNP, PCT, and SIGIRR levels and prognosis of sepsis.

Characteristic	Univariate analysis		Multivariate analysis	
	OR (95% CI)	*P*	OR (95% CI)	*P*
BNP	1.032 (1.010–1.054)	.004	1.023 (1.003–1.042)	.021
PCT	2.495 (1.526–4.081)	<.001	2.928 (1.364–6.286)	.006
SIGIRR	1.083 (1.037–1.131)	<.001	1.126 (1.040–1.220)	.004

BNP = brain natriuretic peptide, CI = confidence interval, OR = odds ratio, PCT = procalcitonin, SIGIRR = recombinant single Ig IL-1-related receptor.

### 3.6. Diagnostic value of serum BNP, PCT, and SIGIRR in prognosis of patients with abdominal infection-related sepsis

**Figure 1.**
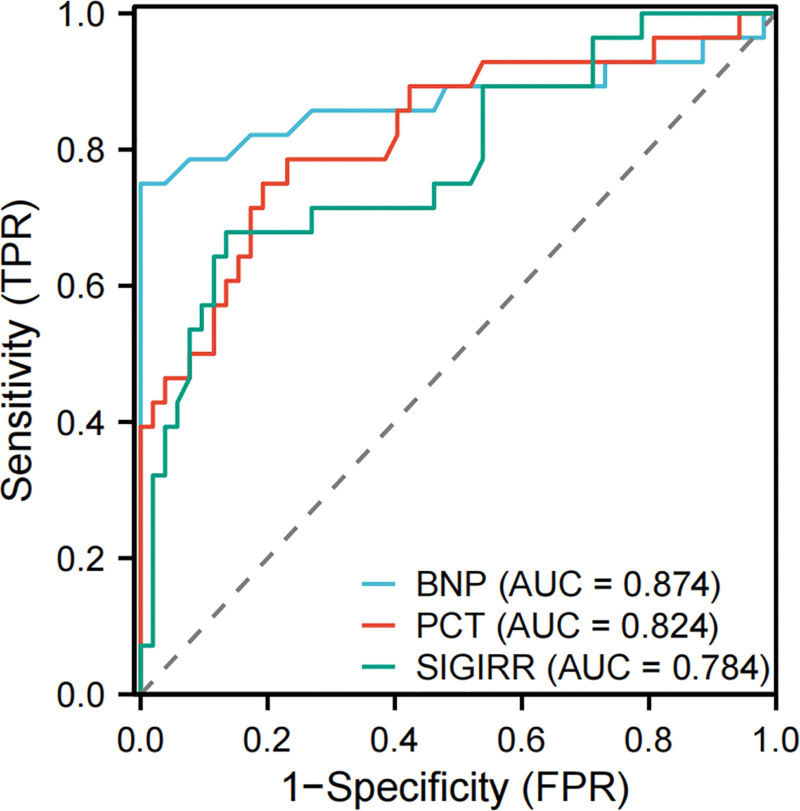
ROC curves of serum BNP, PCT, and SIGIRR in the diagnosis of prognosis of abdominal infection-related sepsis. AUC = area under the curve, BNP = brain natriuretic peptide, FPR = false positive rate, PCT = procalcitonin, ROC = receiver operating characteristic, SIGIRR = recombinant single Ig IL-1-related receptor, TPR = true positive rate.

Taking the death of septic patients within 28 days as the state variable and BNP, PCT, and SIGIRR as the test variables, the ROC curve analysis was performed to evaluate their prognostic diagnostic value. The results showed that the areas under the curve (AUC) of serum BNP, PCT, and SIGIRR in predicting the poor prognosis of septic patients were 0.874 (95% confidence interval [CI] = 0.770–0.978), 0.824 (95% CI = 0.722–0.926), and 0.784 (95% CI = 0.675–0.894), respectively. Based on the Youden index maximum principle, the optimal cutoff values, sensitivity, specificity, prediction accuracy, negative predictive value (NPV), and positive predictive value (PPV) of each indicator were determined as follows: for BNP, the optimal cutoff value was 469.00 pg/mL, with a sensitivity of 75.00%, specificity of 100.00%, prediction accuracy of 91.25%, NPV of 88.14%, and PPV of 100.00%; for PCT, the optimal cutoff value was 8.51 ng/mL, with a sensitivity of 75.00%, specificity of 80.80%, prediction accuracy of 78.75%, NPV of 85.71%, and PPV of 67.74%; and for SIGIRR, the optimal cutoff value was 91.90 pg/mL, with a sensitivity of 67.90%, specificity of 86.50%, prediction accuracy of 80.00%, NPV of 83.33%, and PPV of 73.08. These results suggest that BNP is the strongest predictor of the prognosis of septic patients, followed by PCT, and SIGIRR is the weakest, as shown in Figure 1.

### 3.7. Construction of nomogram prediction model based on serum BNP, PCT, and SIGIRR and its effectiveness evaluation

**Figure 2.**
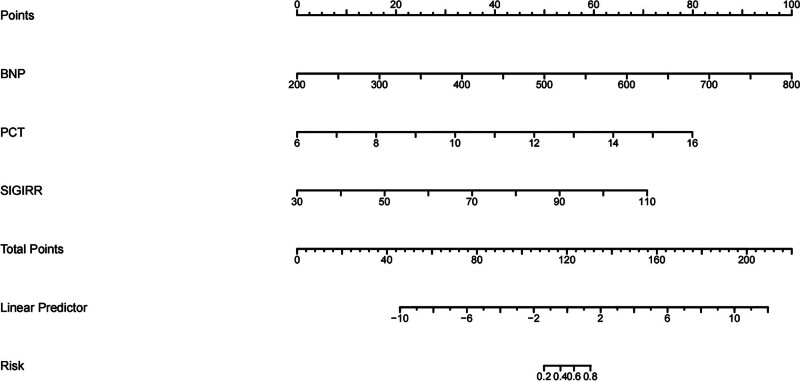
Constructing nomogram of poor prognosis of sepsis patients. BNP = brain natriuretic peptide, PCT = procalcitonin, SIGIRR = recombinant single Ig IL-1-related receptor.

**Figure 3.**
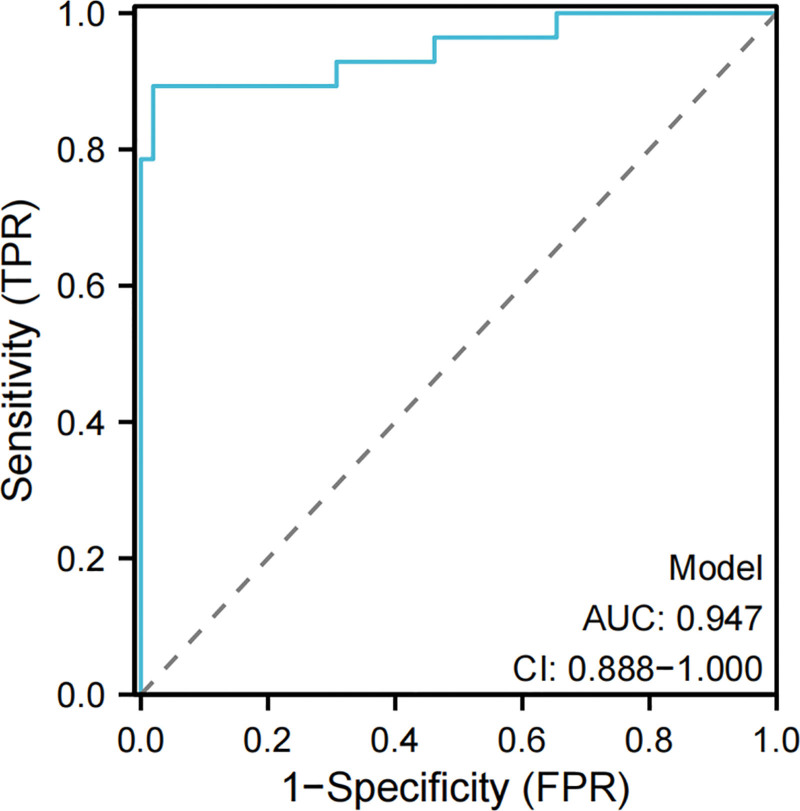
ROC curve for predicting the prognosis of sepsis patients by nomogram. AUC = area under the curve, CI = confidence interval, FPR = false positive rate, ROC = receiver operating characteristic, TPR = true positive rate.

**Figure 4.**
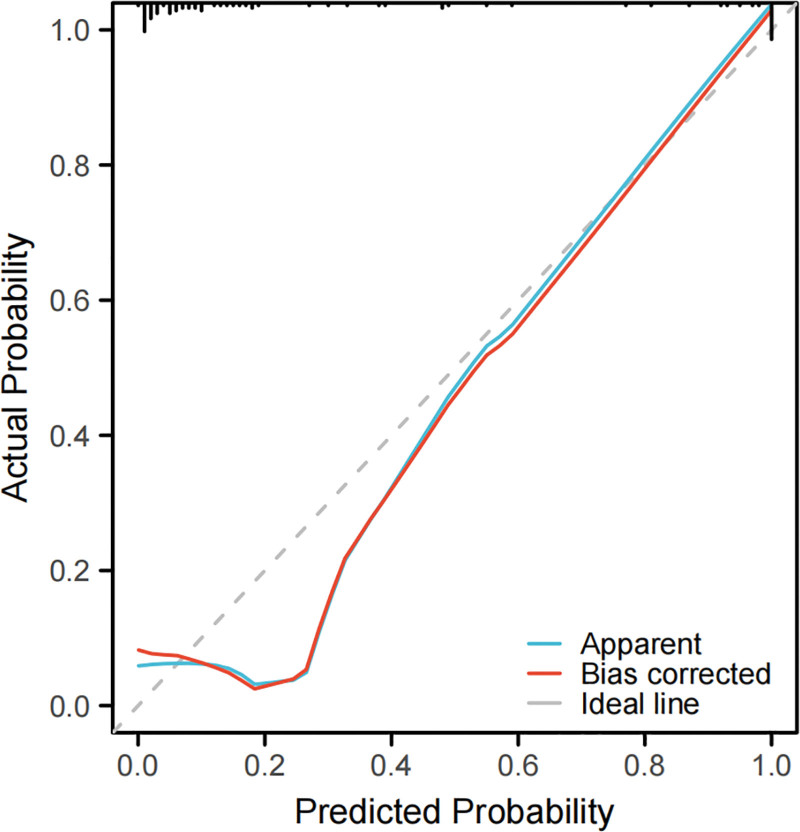
Calibration curve of nomogram for predicting prognosis of sepsis patients.

Based on the independent risk factors (BNP, PCT, and SIGIRR) screened by logistic regression model, the nomogram was constructed, with the C-index of 0.947 and the AUC under ROC curve of 0.947 (95% CI = 0.888–1.000), which indicated that the nomogram had good discrimination and consistency, and the calibration curve showed that the nomogram model predicted the poor prognosis of patients with sepsis associated with abdominal infection and had a high correlation with the actual observation results in the research cohort, as shown in Figures 2, 3, and 4.

## 4. Discussion

According to statistics, the incidence of sepsis in ICU in China is as high as 20.6%, and the mortality of severe sepsis exceeds 50%. Since 2001, Chinese sepsis experts have put forward treatment theories and schemes such as “early target-oriented therapy,” “cluster therapy,” and “restrictive ventilation,” but the incidence and mortality of the disease are still high.^[10]^ In 2018, China’s emergency experts put forward the diagnosis and treatment policy of “early prevention, early detection and early intervention to reduce the morbidity and mortality of sepsis,” confirming the importance of early diagnosis in preventing sepsis and slowing down its progress.^[11]^ Many experts believe that biochemical indicators of infection have certain diagnostic value for emergency judgment of sepsis and its prognosis,^[12]^ and clinical monitoring of inflammatory factors and inflammatory-related substances can provide evidence for clinical treatment of sepsis.

BNP is a neurohormone secreted by the heart, which has the functions of diuresis, sodium excretion, vasodilation, and renin inhibition. The increase of BNP level is mainly caused by the increase of cardiac volume load and vascular smooth muscle proliferation, and it is an important standard to reflect the severity of heart failure.^[13]^ In recent years, many studies have shown that nearly 50% patients with sepsis have symptoms of myocardial function damage within 48 hours after admission. When the ventricular wall is stimulated by stretching and the tension is increased, it can stimulate myocardial cells to secrete pro-B-type natriuretic peptide, which is converted into pro-BNP, and then it is decomposed into active BNP under the action of endonuclease to participate in the biological process of natriuresis and vasodilation.^[14]^ In this study, the expression level of BNP in patients with sepsis associated with abdominal infection and simple abdominal infection was compared. It was found that the serum level of BNP in the sepsis group was significantly higher than that in the sepsis group, and the level of BNP in the death group was significantly higher than that in the survival group in sepsis patients, which indicated that the increase of BNP could indicate the poor prognosis of sepsis. According to the analysis, under normal circumstances, inflammatory factors can mediate the process of tissue damage repair and prevent tissue damage. When the body is infected by a large number of pathogens such as bacteria, the body releases a large number of inflammatory mediators and cytotoxins, such as tumor necrosis factor-α and interleukin, which activate granulocytes, inhibit myocardial contraction, induce mitochondrial membrane damage of myocardial cells, promote the release of oxygen free radicals and lipid metabolites, and then cause inflammatory waterfall reaction. When the structure of myocardial tissue is destroyed, myocardial cells secrete a large amount of BNP precursors, which leads to the increase of BNP level.^[15,16]^ In addition, through the analysis of univariate and multivariate logistics and ROC curves, it is found that BNP is also related to the prognosis of patients with sepsis, suggesting that the increase of BNP is related to the poor prognosis of sepsis and can be used as one of the reference indexes for prognosis evaluation.

PCT is a polypeptide hormone secreted by parafollicular cells, which can reduce blood calcium concentration and inhibit calcium absorption. It is a widely used and studied infection marker so far, and its level can be upregulated after the body is infected by pathogens, and it lasts for a long time.^[17,18]^ In this study, the PCT of all patients was compared, and it was found that the PCT level in the sepsis group was significantly higher than that in the simple infection group, and the PCT in the death group was higher than that in the survival group, suggesting that the increase of PCT can predict the poor prognosis of sepsis. Analysis shows that bacterial endotoxin or bacterial products can stimulate the body to release PCT through Toll-like receptor 4/nuclear factor-k-gene binding when the human body is invaded by bacteria.^[19]^ A meta-analysis of infectious markers shows that the sensitivity and specificity of some traditional markers (such as WBC and CRP) in the diagnosis of infectious diseases are worse than those of PCT.^[20]^ At the same time, there is also evidence that a high level of PCT represents a poor prognosis of patients. Zongxiang et al^[21]^ found that PCT can be used to grade the severity of abdominal infection and help to evaluate the risk of death in hospital. According to the analysis of univariate and multivariate Logistics and ROC curves, PCT is also related to the prognosis of patients with sepsis.

SIGIRR is a superfamily of interleukin receptor-related proteins, which is often expressed in epithelial cells of gastrointestinal tract, kidney, and other organs. SIGIRR has the functions of recognizing interleukin, activating the release of inflammatory bodies, and regulating the inflammatory response of the body.^[22]^ In this study, SIGIRR of all patients was compared, and it was found that the level of SIGIRR in sepsis patients was significantly higher than that in simple infection patients, and the SIGIRR was higher in the death group, suggesting that the increase of SIGIRR was related to the poor prognosis of sepsis. According to the analysis, Toll-like receptor/interleukin-1 receptor signaling pathway can participate in regulating the cell growth of innate immune response and adaptive immune response, and in identifying molecular pathways related to cell injury, thus regulating tissue inflammatory response and body repair. As the receptor of interleukin-1, SIGIRR can trigger the activation of inflammatory corpuscles by recognizing interleukin-1.^[23]^ Related studies have shown that the Toll-like receptor/interleukin-1 receptor signaling pathway regulated by SIGIRR can activate a large number of inflammatory factors and promote the growth and aggregation of macrophages and neutrophils. When a large number of inflammatory factors accumulate in the abdominal cavity or lungs of patients, they play a positive feedback regulation on the body’s own inflammation, thus causing tissue function damage and aggravating the disease.^[24,25]^ In addition, according to the analysis of univariate and multivariate logistics and ROC curves, it was found that SIGIRR was also related to the prognosis of patients with sepsis associated with abdominal infection. The increase of SIGIRR can indicate the aggravation of sepsis, and SIGIRR can be used as one of the clinical reference indexes.

In this study, according to the independent risk factors (BNP, PCT, and SIGIRR) screened by logistic regression model, a nomogram risk prediction model was constructed to predict the poor prognosis of sepsis patients. The nomogram showed strong prediction ability, good discrimination, and consistency (its C-index was 0.947), and we drew a calibration curve, and the results showed that the prediction model had a good fitting degree. The ROC curve shows that the AUC of the nomogram is 0.947, which is significantly higher than that of BNP (AUC = 0.874), PCT (AUC = 0.824), and SIGIRR (AUC = 0.784) in predicting the poor prognosis of septic patients, indicating that the nomogram has higher reference value and is expected to be an effective means to predict the poor prognosis of septic patients.

It is worth noting that the cost of biomarker detection is an important consideration for clinical application, especially in resource-constrained settings. In this study, all detections were performed using ELISA kits, with the approximate cost per patient for BNP, PCT, and SIGIRR being ~$25 to $30, ~$30 to $35, and ~$40 to $45, respectively. The total cost of combined detection of the 3 markers is ~$95 to $110 per patient, which is relatively affordable within the routine clinical testing budget of tertiary hospitals. Timely intervention based on this prediction can reduce the need for invasive treatments, shorten ICU stay (the median ICU stay of the death group was significantly longer than that of the survival group), and lower the overall medical costs associated with septic shock management and organ support. For example, studies have shown that each episode of septic shock increases hospital costs by $10,000 to $20,000; thus, the investment in biomarker detection may yield significant economic returns by preventing disease progression.

The limitations of this study include a relatively small sample size and the lack of a systematic cost-benefit analysis. In future studies, we will expand the sample size to verify the findings in multicenter cohorts and collaborate with health economics researchers to conduct a comprehensive cost-effectiveness evaluation. This will include comparing the cost-benefit ratio of combined detection of BNP/PCT/SIGIRR with that of traditional markers (e.g., WBC, CRP) and evaluating the long-term economic and clinical outcomes of prognostic-guided treatment strategies. Such analyses will further enhance the clinical applicability and promotional value of the findings.

To sum up, BNP, PCT, and SIGIRR are related to patients with sepsis associated with abdominal infection. The nomogram based on BNP, PCT, and SIGIRR has high clinical application value, which can help clinicians to formulate or adjust reasonable diagnosis and treatment plans in time.

## Acknowledgments

The authors would like to thank all colleagues for data collection from the Department of Critical Care Medicine and Department of Gastroenterology, Xining Second People’s Hospital.

## Author contributions

**Data curation:** Yunyun Cui, Jiaqi Yuan, Xiankui Wang, Naixi Ji, Jian Zhang, Changqing Yao.

**Writing – original draft:** Shangzhen Li, Haiyun Zhang.

**Writing – review & editing:** Haiyun Zhang.

## References

[1] Kellum JA Formeck CL Kernan KF Gómez H Carcillo JA. Subtypes and mimics of sepsis. Crit Care Clin. 2022;38:195–211. 35369943 10.1016/j.ccc.2021.11.013 PMC12210553

[2] Srzić I Nesek Adam V Tunjić Pejak D. Sepsis definition: what’s new #8232;in the treatment guidelines. Acta Clin Croat. 2022;61(Suppl 1):67–72. 10.20471/acc.2022.61.s1.11 PMC9536156 36304809

[3] Pierrakos C Velissaris D Bisdorff M Marshall JC Vincent JL. Biomarkers of sepsis: time for a reappraisal. Crit Care. 2020;24:287. 32503670 10.1186/s13054-020-02993-5 PMC7273821

[4] Li N Zhang Y Fan S Xing J Liu H. BNP and NT-proBNP levels in patients with sepsis. Front Biosci (Landmark Ed). 2013;18:1237–43. 23747879 10.2741/4175

[5] Blok DC van Lieshout MH Hoogendijk AJ. Single immunoglobulin interleukin-1 receptor-related molecule impairs host defense during pneumonia and sepsis caused by Streptococcus pneumoniae. J Innate Immun. 2014;6:542–52. 24556793 10.1159/000358239 PMC6741518

[6] Group of Surgical Infection and Critical Care Medicine, Surgery Branch of Chinese Medical Association, Professional Committee of Intestinal Fistula Surgeons Branch of Chinese Medical Association. Guidelines for diagnosis and treatment of abdominal infection in China (2019 Edition). China J Pract Surg. 2020;40:1–16.

[7] Emergency Physician Branch of Chinese Medical Doctor Association, Shock and Sepsis Professional Committee of China Research Hospital Society. Guidelines for emergency treatment of sepsis/septic shock in China (2018). Infect Inflam Repair. 2019;20:3–22.

[8] Bahtouee M Eghbali SS Maleki N Rastgou V Motamed N. Acute physiology and chronic health evaluation II score for the assessment of mortality prediction in the intensive care unit: a single-centre study from Iran. Nurs Crit Care. 2019;24:375–80. 30924584 10.1111/nicc.12401

[9] Pölkki A Pekkarinen PT Takala J Selander T Reinikainen M. Association of sequential organ failure assessment (SOFA) components with mortality. Acta Anaesthesiol Scand. 2022;66:731–41. 35353902 10.1111/aas.14067 PMC9322581

[10] Xiaobin C Gang L Huiling Q. Effects of different doses of Xuebijing injection on inflammatory indexes, alternative treatment and prognosis of sepsis patients. China J Int Trad Chin Western Med. 2023;30:132–5.

[11] Zongxiang Z Mao J Dilong F. Consensus of emergency experts on “Early Prevention and Blockage of Sepsis” in China. Chin Emerg Med Crit Diseases. 2020;32:518–30.

[12] Li H Shan-Shan Z Jian-Qiang K Ling Y Fang L. Predictive value of C-reactive protein and NT-pro-BNP levels in sepsis patients older than 75 years: a prospective, observational study. Aging Clin Exp Res. 2020;32:389–97. 31214930 10.1007/s40520-019-01244-0

[13] Pandompatam G Kashani K Vallabhajosyula S. The role of natriuretic peptides in the management, outcomes and prognosis of sepsis and septic shock. Rev Bras Ter Intensiva. 2019;31:368–78. 31618357 10.5935/0103-507X.20190060 PMC7005946

[14] Großmann S Geisreiter F Schroll S. Natriuretic peptides in intensive care medicine. Med Klin Intensivmed Notfmed. 2023;118:527–33. 37099150 10.1007/s00063-023-01002-1

[15] Yue L Deng X Yang M Li X. Elevated B-type natriuretic peptide (BNP) and soluble thrombomodulin (sTM) indicates severity and poor prognosis of sepsis. Ann Palliat Med. 2021;10:5561–7. 34044565 10.21037/apm-21-1048

[16] Yajing Z Yunjing S Linqing W. Effects of sodium-glucose cotransporter 2 inhibitor combined with novel angiotensin receptor enkephalinase inhibitors on N-terminal pro-brain natriuretic peptide, inflammatory factors and myocardial energy metabolism in patients with type 2 diabetes mellitus complicated with heart failure. China Diabetes J. 2023;31:683–7.

[17] Li Y Min L Zhang X. Usefulness of procalcitonin (PCT), C-reactive protein (CRP), and white blood cell (WBC) levels in the differential diagnosis of acute bacterial, viral, and mycoplasmal respiratory tract infections in children. BMC Pulm Med. 2021;21:386. 34836530 10.1186/s12890-021-01756-4 PMC8620633

[18] Matur E Özcan M Ergül Ekiz E. Use of serum procalcitonin (PCT) level and PCT mRNA expression as a potential clinical biomarker in cats with bacterial and viral infections. J Feline Med Surg. 2022;24:e595–602. 36350675 10.1177/1098612X221125570 PMC10812354

[19] Tominari T Matsumoto C Tanaka Y. Roles of toll-like receptor signaling in inflammatory bone resorption. Biology (Basel). 2024;13:692. 39336119 10.3390/biology13090692 PMC11429252

[20] Tan M Lu Y Jiang H Zhang L. The diagnostic accuracy of procalcitonin and C-reactive protein for sepsis: a systematic review and meta-analysis. J Cell Biochem. 2019;120:5852–9. 30417415 10.1002/jcb.27870

[21] Zongxiang Z Mao J Dilong F. Study on the levels of PCT and TNF-α, CRP and IL-6 in patients with surgical abdominal infection and the degree of infection. Chin J Nosocomiol. 2017;27:4712–5.

[22] Giannoudaki E Stefanska AM Lawler H. SIGIRR negatively regulates IL-36-driven psoriasiform inflammation and neutrophil infiltration in the skin. J Immunol. 2021;207:651–60. Epub 2021 Jul 12. Erratum in: J Immunol. 2021;207:2895. doi: 10.4049/jimmunol.2100907. 34253575 10.4049/jimmunol.2100237

[23] Ye Y Shi Y Wei Z Liu H Li W. SIGIRR suppresses hepatitis B virus X protein-induced chronic inflammation in hepatocytes. Gene. 2024;928:148768. 39013482 10.1016/j.gene.2024.148768

[24] Li L Wei J Suber TL. IL-37-induced activation of glycogen synthase kinase 3β promotes IL-1R8/Sigirr phosphorylation, internalization, and degradation in lung epithelial cells. J Cell Physiol. 2021;236:5676–85. 33400290 10.1002/jcp.30253 PMC8809512

[25] Li L Wei J Li S. The deubiquitinase USP13 stabilizes the anti-inflammatory receptor IL-1R8/Sigirr to suppress lung inflammation. EBioMedicine. 2019;45:553–62. 31204278 10.1016/j.ebiom.2019.06.011 PMC6642080

